# Within-session dose–response and between-session carry-over effects of eccentric contractions versus static stretches on range of motion and muscle–tendon mechanics

**DOI:** 10.1007/s00421-025-05979-9

**Published:** 2025-09-18

**Authors:** Anthony David Kay, Anthony William Baross, Brett Anthony Baxter, Anthony John Blazevich

**Affiliations:** 1 Centre for Physical Activity and Life Sciences, Faculty of Art, Science and Technology, University of Northampton, Northamptonshire, NN1 5PH UK; 2 Centre for Human Performance (CHP), School of Medical and Health Sciences, Edith Cowan University, Joondalup, Australia

**Keywords:** Flexibility, ROM, Eccentric exercise, Tissue stiffness, Stretch tolerance

## Abstract

**Purpose:**

Eccentric resistance training produces substantial increases in maximum joint range of motion (ROM), highlighting its potential as an alternative to static stretching. However, little is known about the short-term effects or associated mechanisms. Therefore, this study compared within-session responses and between-session carry-over effects of eccentric contractions and static stretching in 18 healthy human volunteers.

**Methods:**

Using a counterbalanced crossover design, participants completed four sessions across two conditions: Eccentric contractions (EC_1_, EC_2_) and static stretching (SS_1_, SS_2_), with 48–72 h between sessions. EC comprised 5 sets of 10 × 3-s isokinetic eccentric contractions while SS comprised 5 sets of 30-s static stretches (total time under tension = 150 s). Dorsiflexion ROM and passive plantarflexor torque were recorded before and after each set, and maximal isometric plantarflexor torque, active Achilles tendon stiffness, and passive gastrocnemius medialis stiffness were measured pre- and post-intervention.

**Results:**

Significant within-session increases in ROM (2.2°–6.0°) and reductions in muscle–tendon unit (MTU) stiffness (2.7–7.3%) and muscle stiffness (8.4%) occurred after both EC_1_ and SS_1_. However, only EC_1_ improved stretch tolerance (30.7%) and decreased Achilles tendon stiffness (12.0%). Comparable between-session carry-over effects occurred after two sessions of stretches and contractions in pre-intervention ROM (5.9°, collapsed data), stretch tolerance (38.0%), and MTU stiffness (41.7%).

**Conclusion:**

Eccentric contractions produced more than twice the acute ROM increase and greater changes in stiffness and stretch tolerance than static stretching. The significant carry-over effects after two sessions also indicate a more potent stimulus for increasing ROM, with important implications for clinical practice and exercise prescription.

## Introduction

Limited joint range of motion (ROM) and greater resistance to stretch within the ROM (i.e. tissue stiffness) are associated with increased muscle strain injury risk (Witvrouw et al. 2004; Watsford et al. 2010) and can negatively influence performances in activities of daily living (Mulholland and Wyss 2001; Spink et al. 2011). A range of muscle stretching exercises (e.g. static, dynamic, ballistic, proprioceptive neuromuscular facilitation [PNF]) have been utilised to increase joint ROM (Babault et al. 2010; Li et al. 2020), with passive static stretching being the most common in athletic (Babault et al. 2021) and clinical (Gomez-Cuaresma et al. 2021) environments. The efficacy of these stretching techniques to promote chronic increases in ROM has been confirmed in healthy populations in several systematic reviews (Behm et al. 2016; Medeiros and Lima 2017; Cayco et al. 2019). However, stretching often fails to provide meaningful improvements in a range of clinical conditions (Harvey et al. 2017), where ROM is often compromised by neurological impairments resulting in substantial maladaptation to muscle structure and function (i.e. spasticity, contracture, hemiparesis). This inability to improve ROM may be a consequence of stretch-induced gains in ROM being more commonly associated with an increase in ‘stretch tolerance’ (i.e. a reduced apprehension, discomfort, or pain perception) rather than changes in mechanical properties (i.e. reduced muscle stiffness) or structural characteristics (i.e. muscle architecture) (Weppler and Magnusson 2010; Konrad and Tilp 2014; Blazevich 2019). Therefore, identifying alternative interventions that result in large acute and chronic increases in ROM, whilst simultaneously influencing important muscle mechanical properties and functional characteristics, may have more meaningful athletic and clinical relevance.

Recent reviews have confirmed consistent increases in joint ROM following longer-term eccentric resistance training programmes (O’Sullivan et al. 2012; Vetter et al. 2022; Diong et al. 2022; Kay et al. 2023), with a recent meta-analysis (Kay et al. 2023) reporting a large effect (*g* = 0.86) on passive lower-limb ROM that was substantially greater than the small effects (*g* = 0.29–0.49) reported in another meta-analysis following traditional resistance training or muscle stretching programmes (Afonso et al. 2021). Whilst the efficacy of eccentric training programmes to promote longer-term increases in ROM has been confirmed, limited data exist describing its acute effects, with only six studies being published focusing on the acute effect of eccentric contractions on ROM (Nelson 2006; Nishida et al. 2018; Aune et al. 2019; Kawama et al. 2022, 2023; Muanjai et al. 2022). Nonetheless, consistent with the meta-analyses examining the chronic effects, these studies reported significant increases in hip flexion and dorsiflexion ROM, highlighting the potential of eccentric contractions as an alternative exercise to acutely increase ROM. However, no information exists on the cumulative within-session dose–response characteristics, or residual between-session carry-over effects that might ultimately manifest in longer-term chronic changes to ‘permanently’ enhance ROM, nor how quickly (i.e. over how many exercise sessions) these changes might manifest. Consequently, there is a need to determine the dose–response and carry-over effects of eccentric contractions to identify whether this intervention can be used as an alternative to current muscle stretching practice.

Joint ROM is influenced by neural (e.g. stretch tolerance, pain perception), mechanical (e.g. tissue stiffness), and structural (e.g. muscle architectural) factors (Weppler and Magnusson 2010). Since improvements might have both psychobiological (tolerance) and mechanical (tissue stiffness) underpinnings, and because eccentric contractions impose a greater load than static stretching offering substantially greater sensory and mechanical stimuli, there may be potential for greater acute increases following eccentric contractions. Whilst there is substantial evidence that acute stretch-induced increases in ROM are primarily associated with increased stretch tolerance and decreased muscle tissue stiffness, only four studies to our knowledge have examined the mechanisms associated with ROM changes following eccentric knee flexor (Nishida et al. 2018; Kawama et al. 2022, 2023; Muanjai et al. 2022) or plantarflexor (Muanjai et al. 2022) contractions. No change in stretch tolerance (i.e. peak passive torque (Nishida et al. 2018; Kawama et al. 2022, 2023)) or MTU stiffness (assessed via the stress–strain model (Muanjai et al. 2022)) were detected in these studies, with inconsistent reductions in shear modulus (indicative of static [but not dynamic] muscle stiffness) between individual hamstring muscles, assessed via shear wave elastography (Kawama et al. 2022, 2023)). Given the paucity of literature, disparate measurement techniques, and inconsistent findings, further research is needed to fully elucidate the mechanisms associated with acute increases in ROM following eccentric contractions.

Given the above, the aims of the present study were to examine and compare the novel within-session dose–response effects (i.e. examined after the first exposure of passive, static stretches and eccentric contractions) and between-session carry-over effects after both one (i.e. determined before the second session) and two (i.e. determined before the third session) sessions of passive static stretching or eccentric contractions. Outcome variables included dorsiflexion ROM, peak passive plantarflexor torque (i.e. stretch tolerance), the slope of the passive torque curve (i.e. MTU stiffness), passive gastrocnemius medialis (GM) muscle stiffness, active Achilles tendon stiffness, and maximal isometric plantarflexor torque. We tested the hypotheses that such exercises would result in (i) significant within-session (i.e. between sets) increases in ROM and stretch tolerance alongside significant decreases in MTU stiffness, tendon stiffness (eccentric contractions only), and maximal isometric torque (static stretching only), (ii) significantly greater within-session changes in ROM with eccentric contractions, and (iii) a significant difference in stretch behaviour and muscle–tendon properties measured before each of two subsequent sessions (i.e. a residual between-session carry-over effect detected at the beginning of Sessions 2 and 3) only after the eccentric contractions.

## Materials and methods

### Participants

Twenty recreationally active participants with no recent history (≥ 2 years) of lower-limb injury volunteered for the study after completing a pre-test medical questionnaire and providing written and informed consent with 18 (12 females, 6 males; age = 19.8 ± 1.3 years, height = 1.7 ± 0.1 m, mass = 71.1 ± 8.8 kg) completing the study. Participants were undergraduate Sport Science students that were recreational gym users and/or played weekly in a range of team sports. All participants were familiar with static stretching and resistance training that included eccentric muscle actions, however all participants were naive to isokinetic eccentric muscle actions. Ethical approval was granted by the Ethics Committee at the University of Northampton, with the study completed in accordance with the Declaration of Helsinki.

### Procedures

#### Overview

**Fig. 1:**
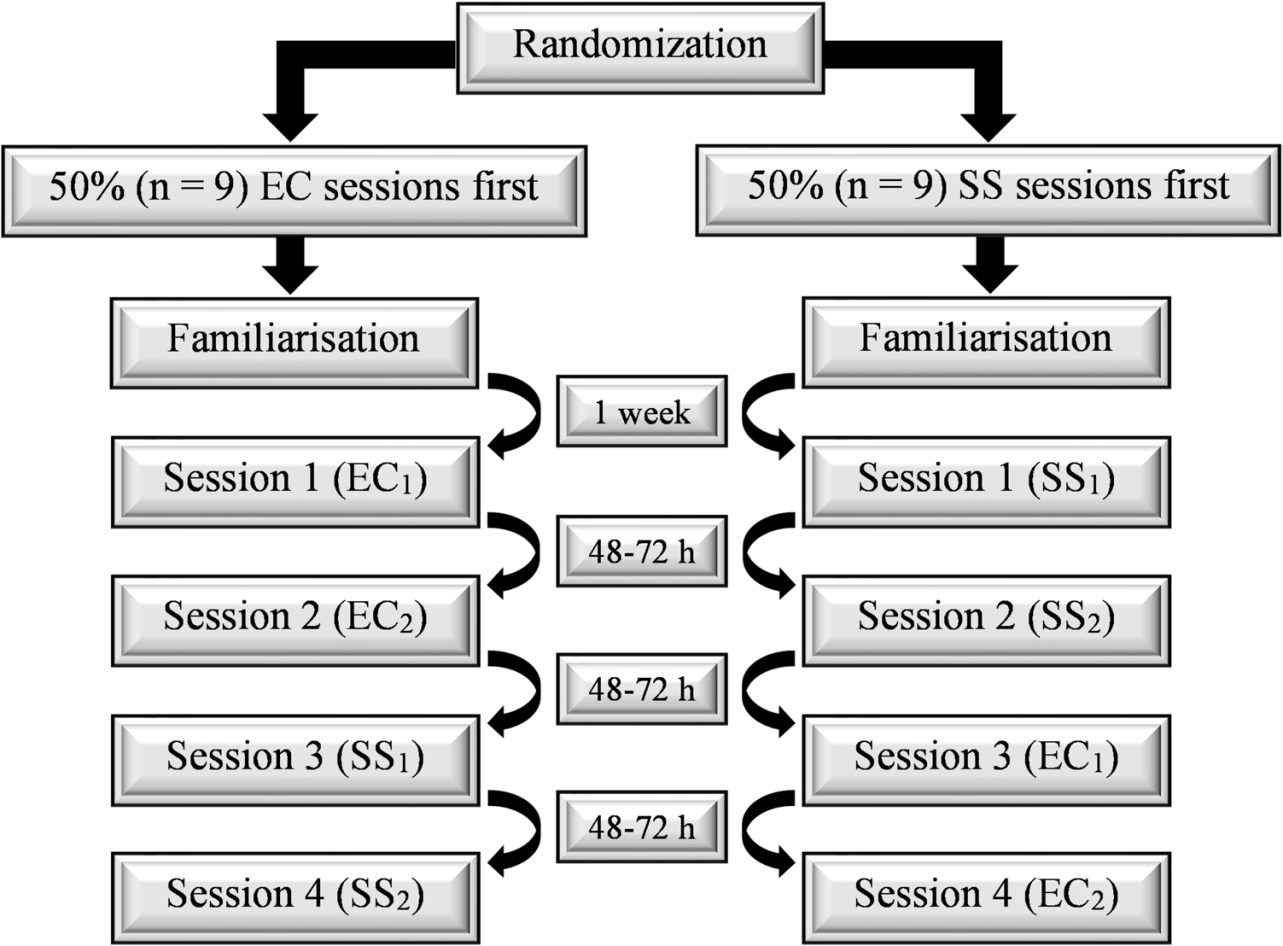
Schematic showing randomised counterbalanced crossover study design. Participants were initially randomised into two groups (counterbalanced by intervention order) before visiting the laboratory for familiarisation. Subsequently, participants completed four sessions under two experimental conditions (eccentric contractions [EC] and static stretching [SS]) with each condition performed on two separate occasions with each session separated by 48–72 h. Fifty percent of participants performed the four sessions in the following order: Session 1 (eccentric contractions [EC_1_]), Session 2 (eccentric contractions [EC_2_]), Session 3 (static stretching [SS_1_]), Session 4 (SS_2_]). The remaining 50% performed the four sessions in reverse order: Session 1 (static stretching [SS_1_]), Session 2 (static stretching [SS_2_]), Session 3 (eccentric contractions [EC_1_]), Session 4 (eccentric contractions [EC_2_])

The participants were familiarised with the experimental testing protocols one week prior to data collection. As the aims of the study were to determine acute and carry-over effects, familiarisation consisted of repeated passive ROM assessments until consistent ROM (within 3°) was achieved, followed by a short exposure to muscle stretching (single 30 s stretch) to ensure the participants were accustomed to the stretch in a dynamometry setting. This was followed by participants performing 4–6 submaximal (~ 50% of perceived maximum) eccentric contractions to ensure participants were able to consistently perform the eccentric contraction during dorsiflexion and relax as the dynamometer returned the participant to the starting position. Participants then performed 3 repetitions at maximal effect, with the brief exposure to these contractions implemented to mitigate potential repeated bout effects from influencing outcomes in subsequent testing sessions. They then visited the laboratory for four further sessions under two experimental conditions (eccentric contractions [EC_1_, EC_2_] or static stretching [SS_1_, SS_2_]) using a randomised, counterbalanced crossover design with 50% (*n* = 9) performing the four sessions in the order EC_1_, EC_2_, SS_1_, SS_2_, with the other 50% performing in the order SS_1_, SS_2_, EC_1_, EC_2_; each session was separated by 48–72 h (Fig. 1).

During each session, the participants performed a 5-min jogging warm-up on a treadmill at a slow but self-selected pace at the transition between walking and jogging (2.02 ± 0.17 m s^−1^). Participants were then seated in the chair of an isokinetic dynamometer (Biodex System 4 Pro, IPRS, Suffolk, UK) with the hip at 70° (0° = full extension), right knee fully extended (0°) and foot positioned in the dynamometer footplate with the lateral malleolus aligned with the centre of rotation of the dynamometer and the sole of the foot perpendicular to the shank to ensure the ankle was in the anatomical position (0°). To ensure valid and reliable ROM and torque data, heel displacement from the footplate was minimised with a rachet mechanism utilising non-elastic strapping across the ankle. The leg ‘lock-out’ method was also employed with the participants’ knee slightly flexed before locking out the knee with non-elastic strapping placed across the thigh to maintain knee extension, which presses the pelvis into the backrest of the seat to further minimise lateral movement and heel displacement (Cannavan et al. 2012). One experienced analyst conducted all maximal voluntary isometric contraction (MVIC) and ROM tests to remove inter-tester variability, with test re-test reliabilities determined using intraclass correlation coefficients with 95% confidence intervals (CI) and coefficients of variation (CV) that revealed good-to-excellent reliability for MVIC (ICC = 0.89 [CI 0.76, 0.96]; CV = 12.4%) and ROM (ICC = 0.97 [CI 0.93, 0.99]; CV = 4.4%) of these methods in our laboratory reported previously (Kay et al. 2015).

The participants initially performed two submaximal isometric plantarflexor contractions in the anatomical position (0°) at 50% and 75% perceived maximal intensity with 15 s rest between contractions, before performing an MVIC to enable maximal isometric plantarflexor torque and Achilles tendon stiffness to be calculated (described below), with identical methods employed to minimise heel displacement as described earlier in the passive ROM tests. One minute later, the participants performed three passive ROM tests to quantify dorsiflexion ROM, peak passive torque (stretch tolerance), passive MTU stiffness (slope of the passive torque curve), and passive GM muscle stiffness (described below). The participants then performed either five sets of 10 × 3-s eccentric contractions (EC) or five sets of 30-s static stretches (SS). Three passive ROM tests were then performed after each set to determine the within-session dose–response effect. After the final ROM test, participants performed an MVIC to determine the impact of the 5 sets of contractions or stretches on maximal isometric plantarflexor torque and active tendon stiffness (described below).

#### Range of motion and passive torque

**Fig. 2:**
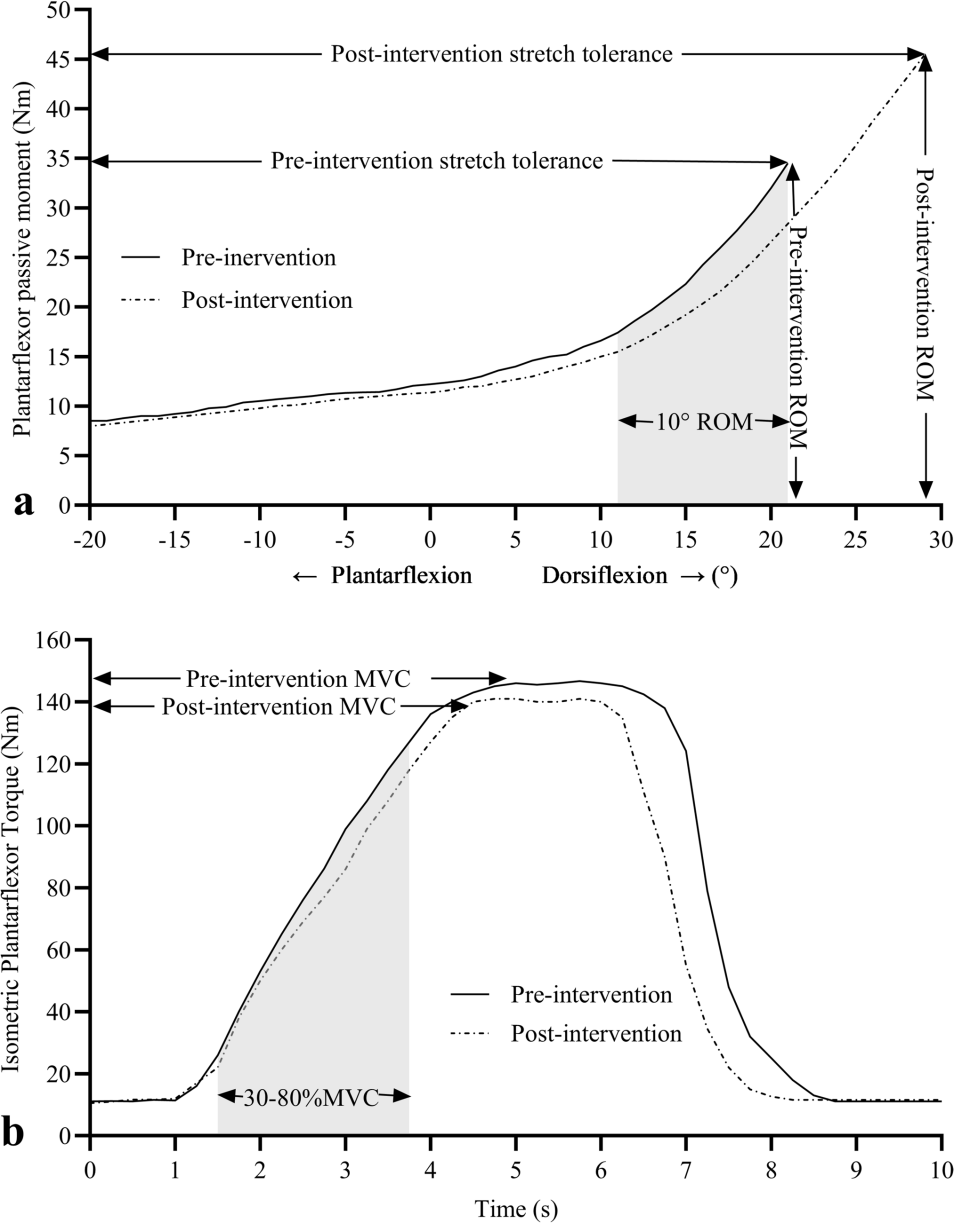
Passive (**a**) and isometric (**b**) plantarflexor torques recorded during pre- and post-intervention testing (one subject’s data depicted during an eccentric trial). Range of motion, peak passive moment (stretch tolerance), and the slope of the passive moment curve (calculated in the final 10° of ROM [shaded area]) were measured during the third passive ROM trial before and after each set of contractions or stretches (pre- and post-intervention traces depicted only to aid clarity). Maximal isometric moment and Achilles tendon stiffness (measured from 30–80%MVC [shaded area]) were recorded during a ramped maximal isometric contraction performed before and after the interventions

To determine the within-session dose–response effect, data collected in the first session of eccentric contractions (i.e. EC_1_) and static stretches (i.e. SS_1_) were used. Before and then after each set of contractions or stretches, three ROM tests were performed at 30-s intervals and data were recorded from the third test to minimise the potential for thixotropic properties of skeletal muscle to influence the torque data (Proske and Morgan 1999). The participant’s foot was passively dorsiflexed from 20° plantarflexion through to the greatest volitional dorsiflexion angle at 0.087 rad s^−1^ (5° s^−1^) where they terminated the movement by pressing a hand-held release button at the point of discomfort; a stretch intensity commonly used in ROM studies (Kay et al. 2015). The movement velocity was chosen as it is too slow to elicit a significant myotatic stretch reflex response (McNair et al. 2001), ensuring that full volitional ROM was reached and that the torque data were reflective of the passive plantarflexor properties. ROM (°), peak passive torque (i.e., stretch tolerance [Nm]; measured within 250 ms of peak dorsiflexion), and the slope of the passive torque–angle relation (i.e., MTU stiffness [Nm°^−1^]; calculated as the change in plantarflexor torque through the final 10° of dorsiflexion) were measured using an identical joint angle range after each set to ensure the same region of the torque–angle curve was measured (Fig. 2a) and did not influence MTU stiffness data (Kay et al. 2015). During all tests, plantarflexor torque and dorsiflexion ROM data were directed from the dynamometer to a high-level transducer (model HLT100C, Biopac, Goleta, CA) before analog-to-digital conversion at a 2000-Hz sampling rate (model MP150 Data Acquisition, Biopac). The data were then directed to a personal computer running AcqKnowledge software (v5.0, Biopac) and filtered with a zero lag, 6-Hz Butterworth low-pass filter prior to plantarflexor torques being determined.

#### Muscle activity

To confirm minimal muscle activity during ROM assessments, skin-mounted bipolar double differential active electrodes (model MP-2A, Linton, Norfolk, UK) were placed over the medial aspect of the soleus muscle with a reference (ground) electrode over the tibia. EMG data were amplified (gain = 300, input impedance = 10 GΩ, common mode rejection ratio ≥ 100 dB at 65 Hz) and directed to a high-level transducer (model HLT100C, Biopac) before analog-to-digital conversion at a 2000-Hz sampling rate (model MP150 Data Acquisition, Biopac). The data were stored on a personal computer running AcqKnowledge software (v5.0, Biopac) and processed using a 20- to 500-Hz band-pass filter and converted to root-mean-squared EMG with a moving 250-ms averaging window. The EMG data were then normalised as a percentage of the peak amplitude recorded during the pre-intervention MVIC (described later). The normalised EMG amplitude (%MVC) was used as a measure of neuromuscular activity, which was then quantified within a 250-ms epoch at full ROM.

#### Passive gastrocnemius medialis muscle stiffness

The position (i.e. excursion) of the GM-Achilles muscle–tendon junction (MTJ) was recorded during the passive and active tests using real-time B-mode ultrasound imaging (Vivid I, General Electric, Bedford, UK) using a scanning frequency of 10 MHz with video recorded at 28 Hz using a wide-band linear probe (8L-RS, General Electric) with a 39-mm field of view. The probe was placed within a custom-made high-density foam housing and manipulated until the superficial and deep GM aponeuroses could be visualised to enable triangulation of the GM-Achilles MTJ. The housing was then fixed to the skin with zinc-oxide adhesive tape to ensure consistent imaging of the MTJ. The distance between the MTJ and distal edge of the ultrasound image was manually digitised (Vivid I, General Electric) in each image during passive (final 10° of ROM) and active (30–80%MVIC) tests to enable the change in GM muscle and Achilles tendon lengths to be calculated. Intratester reliability for the manual digitisation of MTJ excursion within the ultrasound images (*n* = 5) has been determined previously in our laboratory (Kay and Blazevich 2009) by calculating the ICC (95% CI) and CV. A high ICC (0.99 [CI = 0.98, 1.00]) and low CV (0.4%) were calculated with no significant difference detected between mean values (*P* > 0.05), indicating excellent reliability.

Plantarflexor torque and ultrasound data were electronically synchronised using a 5-V ascending transistor-transistor logic (TTL) pulse that simultaneously ended the capture of ultrasound data (preceding 15 s of data) and placed a marker on the torque data record (i.e. the final frame of the ultrasound video corresponded with the marker on the torque-time trace). Passive muscle stiffness was calculated as the change in torque through the final 10° of dorsiflexion (i.e. in the linear portion of the stress–strain curve) divided by the change in muscle length (Nm mm^−1^), with an identical joint angle range used in post-intervention tests.

#### Maximal isometric plantarflexor torque

Quantification of the within-session dose–response effect on maximal plantarflexor torque was not possible as repeated MVICs before and after each set would influence tendon stiffness (Obst et al. 2013) and mask the potential effects of the EC and SS interventions. However, the effect of the (five sets of) eccentric contractions and static stretches was measured using single 5-s ramped MVICs performed before and after the interventions with the ankle in the anatomical position (0°). Contraction intensity was gradually increased linearly over time with maximal plantarflexor torque reached after ~ 3 s and then held for 2 s, enabling a visible plateau in the torque-time trace to confirm maximal isometric plantarflexor torque was achieved (Fig. 2b).

#### Active Achilles tendon stiffness

The ~ 3-s ramped MVIC also enabled tendon elongation to be recorded using sonography, which allowed tendon stiffness to be calculated when combined with plantarflexor torque data (Kay and Blazevich 2009). During familiarisation, ramped contractions were repeated using visual feedback until the participants could reliably achieve a linear increase in plantarflexor torque and reach MVIC after ~ 3 s to minimise the potential for loading rate to influence tendon stiffness (Seynnes et al. 2015). While greater heel displacement from MVICs could influence the accuracy of tendon length calculations, excellent within-session reliability for tendon length (ICC = 0.99 [CI 0.98, 1.00]; CV = 1.0%) has been demonstrated previously (Kay and Blazevich 2009). Active tendon stiffness was then calculated during the MVIC tests as the change in plantarflexor torque from 30–80%MVIC divided by the change in tendon length (Nm mm^−1^). The interval time taken to increase plantarflexor torque through 30–80% of MVIC (i.e. the loading range over which tendon stiffness was calculated) was recorded pre- and post-intervention, with no significant difference (pre-intervention = 1.52 ± 0.62 s, post-intervention = 1.80 ± 0.65 s) detected, indicating that similar strain rates were achieved.

#### Eccentric contraction intervention

During familiarisation, maximal isometric torque was determined at 20° plantar flexion (i.e. initiation point of the eccentric contractions) with the dynamometer programmed to begin ankle dorsiflexion only when torque reached 80%MVIC; this ensured participants were at near-maximal effort before initiating the rotation of the dynamometer to enable maximal eccentric plantarflexor contractions. Participants performed five sets of isokinetic eccentric plantarflexor contractions (10 repetitions per set) with each contraction performed through a submaximal ROM that was initiated from 20° plantarflexion though to 10° dorsiflexion at 10° s^−1^ resulting in a 3-s contraction (total time under tension = 150 s). The submaximal joint angle range ensured all participants contracted through the same joint angles, removing the potential for contractions being performed at different muscle lengths from influencing the outcome measure. This protocol has been used previously with large increases in ROM detected after a six-week protocol (Kay et al. 2016), thus we were confident of the ability of the submaximal contraction range to induce increases in ROM. The participants then relaxed as the dynamometer returned the ankle to the starting position at 30° s^−1^, which provided a 1-s rest between contractions. Each set was separated by 2 min to enable three passive ROM tests to be performed to determine the cumulative effects of each successive set within the session.

#### Static stretch intervention

The participants performed five sets static stretches (30 s per set) resulting in a total of 150 s of static stretch. Each stretch was initiated from 20° plantarflexion with the ankle passively dorsiflexed at 5° s^−1^ through their full ROM until they pressed a hand-held release button at the point of discomfort to volitionally terminate the movement. The participants were instructed to remain relaxed throughout the rotation and static stretch, and the dynamometer returned the participant to 20° plantarflexion after 30 s of static stretch. Identical to the eccentric intervention, two minutes were provided between each set to enable three passive ROM tests to be performed to determine the impact of each successive stretch within the session.

### Data analyses

All data were analysed using the Statistical Package for the Social Sciences (SPSS) software (v28; IBM, Chicago, IL) and reported as mean ± SD. Normal distribution for all data sets was assessed using Shapiro–Wilk tests with Levene’s test used to determine homogeneity of variance. Where normal distribution was violated, successful log transformations (log_10_) were performed for peak passive torque and MTU stiffness. To test for within-session dose–response effects (where participants were naïve to the interventions), data from the first sessions (i.e. EC_1_ and SS_1_) were examined using separate two-way repeated measures ANOVAs to test for the effects of condition (× 2 [EC, SS]) and time (× 6 [pre-intervention, 30 s [set 1], 60 s [set 2], 90 s [set 3], 120 s [set 4], 150 s [set 5]) in (i) dorsiflexion ROM, (ii) soleus EMG (muscle activity), (iii) peak passive torque (stretch tolerance), and (iv) slope of the passive torque curve (MTU stiffness). Two-way repeated measures ANOVAs were also used to test for the within-session effects of condition (× 2 [EC, SS) and time (× 2 [pre-intervention, post-intervention) in (v) passive gastrocnemius medialis (GM) muscle stiffness, (vi) active Achilles tendon stiffness, and (vii) maximal isometric plantarflexor torque.

To test for between-session carry-over effects (i.e. whether one or two sessions of eccentric contractions or static stretches were sufficient to alter baseline characteristics at the next session), pre-session data were compared between Sessions 1, 2, and 3. Separate two-way mixed-model ANOVAs were used to test for the between-session carry-over effects of group (× 2 [EC, SS]) and within-session effects of time (× 3 [Session 1, Session 2, Session 3]) in pre-intervention (i) dorsiflexion ROM, (ii) peak passive torque, (iii) MTU stiffness, (iv) passive GM muscle stiffness, (v) active Achilles tendon stiffness, and (vi) maximal isometric plantarflexor torque. Partial eta squared (*η*_*p*_^2^*)* and Cohen’s d (*d*) were used to calculate effect sizes for ANOVA and *t*-tests, respectively. Pearson’s (*r*) or Spearman’s (*r*_*s*_) correlation tests were conducted to quantify the relationship between changes in all variables. Statistical significance for all tests was accepted at *P* < 0.05.

### Reliability

Test–retest reliability was assessed pre-intervention across the four sessions for ROM, peak passive torque, MTU stiffness, GM muscle and Achilles tendon stiffness, and peak isometric torque. No significant differences were detected between test–retest mean values (*P* > 0.05) for any measure; with high-to-excellent ICCs and low-to-moderate CVs calculated for ROM (ICC = 0.94 [CI 0.88, 0.98, CV = 8.1%), stretch tolerance (ICC = 0.92 [CI 0.84, 0.97], CV = 20.5%), MTU stiffness (ICC = 0.95 [CI 0.90, 0.98, CV = 22.2%), GM muscle stiffness (ICC = 0.91 [CI 0.81, 0.96], CV = 20.5%), Achilles tendon stiffness (ICC = 0.97 [CI 0.93, 0.99], CV = 11.3%), and peak isometric torque (ICC = 0.97 [CI 0.95, 0.99], CV = 8.3%).

### Sample size

Based on data from the only study to directly compare static stretching and eccentric contraction interventions (Nelson 2006), large effect sizes (1.22–1.40 [Cohen’s *d*]) were calculated from mean changes in ROM with a large interaction effect also detected (*η*^2^ = 0.42). A priori power analysis was conducted using G*Power version 3.1.9.7 (Faul et al. 2007) for sample size estimation required to detect significant differences using the following parameters: 1 − β error (power) = 0.80, α = 0.05, effect size (Cohen’s *F*) = 0.40. The power analysis revealed a minimum of 16 participants would be needed with 20 participants recruited to account for possible data loss and participant attrition. Two participants withdrew from the study before completing the four sessions with non-related injuries, with statistical analyses conducted on data sets for 18 participants who completed the testing.

## Results

### Within-session dose–response effects

#### Range of motion

**Fig. 3:**
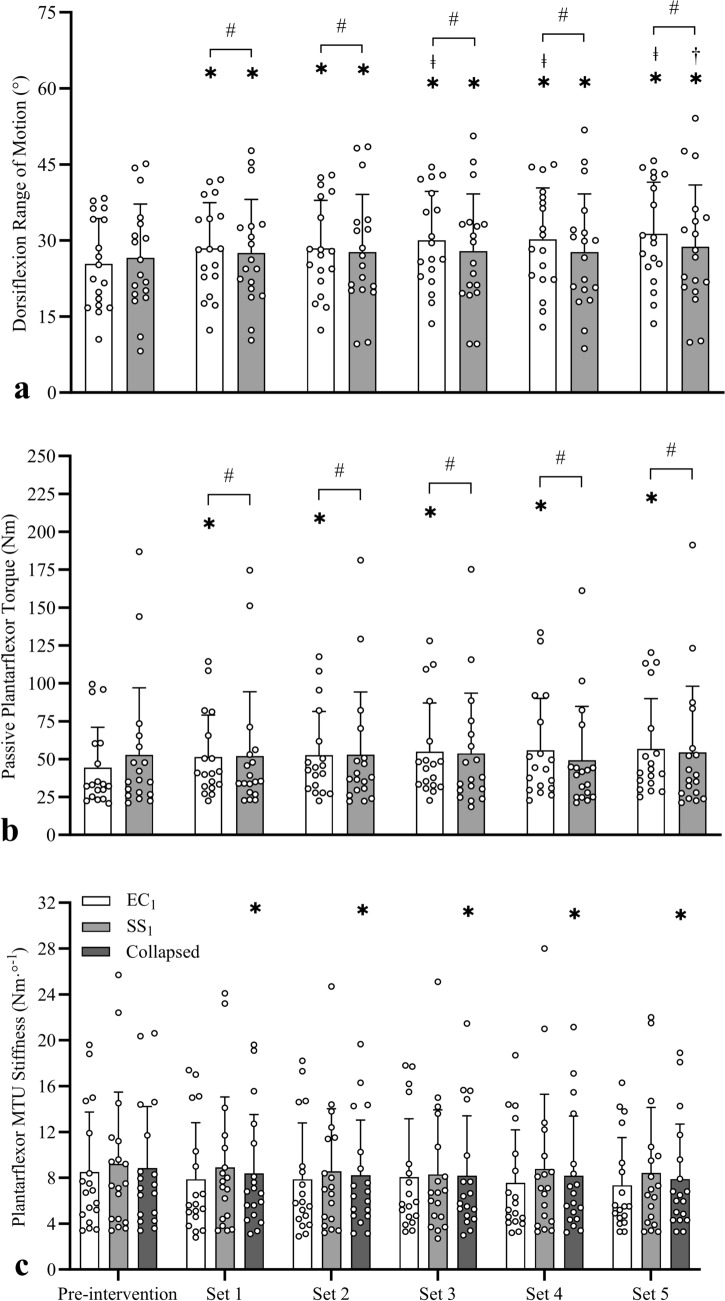
Mean (± SD) and individual dorsiflexion range of motion (**a**), plantarflexor stretch tolerance (**b**), and muscle–tendon unit (MTU) stiffness (**c**) data measured pre- and post-intervention to detect acute effects during the first eccentric contraction (EC_1_) and static stretching (SS_1_) sessions. Compared to pre-intervention, significant increases were detected in range of motion in both conditions, in stretch tolerance only after EC_1_, and reductions in MTU stiffness only when data were collapsed. *Significant within-condition difference to pre-intervention, ^ǂ^Significant within-condition difference to sets 2 and 3, ^†^Significant within-condition difference to set 4, ^#^Significant between-condition difference at the same timepoint (using baseline ANCOVA adjusted analyses)

To examine the within-session dose–response effects, the first sessions in each condition (i.e. EC_1_ and SS_1_) were examined. A two-way repeated measures ANOVA revealed a significant interaction effect for ROM (*F*_3.85,65.44_ = 6.810, *P* < 0.001, *η*_*p*_^2^ = 0.286). Simple main effects analyses revealed significant increases in ROM (Fig. 3a) after each set in EC_1_ (3.1°–6.0°, *d* = 1.04–2.00) and SS_1_ (1.0°–2.2°, *d* = 0.54–0.76) when compared to pre-intervention. In EC_1_, further significant increases in ROM occurred after the 3-5th sets when compared to after the 1st (1.6°–2.9°, *d* = 0.68–1.01) and 2nd (1.6°–2.9°, *d* = 0.59–0.77) sets, indicating that improvements occurred after each set. However, in SS_1_, a further significant increase in ROM was only apparent after the 5th set compared to the 4th (1.0° ± 1.9°, *d* = 0.53). Despite the significant interaction effect, no significant between-condition difference was detected at any timepoint (0.9°–2.6°, *d* = 0.01–0.23), indicative of a crossover profile (i.e. mean ROM pre-intervention was lower in the eccentric group but following the final set it was greater). Consequently, data were re-examined using a baseline-adjusted ANCOVA, which revealed significantly greater ROM in EC_1_ than SS_1_ after each set (1.9°–3.9°, *d* = 0.75–1.53).

#### Soleus muscle activity

No significant interaction effect (*F*_2.99,35.91_ = 0.710, *P* = 0.552, *η*_*p*_^2^ = 0.056) or main effects of condition (*F*_1,12_ = 1.824, *P* = 0.202, *η*_*p*_^2^ = 0.132) or time (*F*_5,60_ = 0.535, *P* = 0.591, *η*_*p*_^2^ = 0.032) were detected for soleus muscle activity, with mean activity remaining below 5%MVC.

#### Peak passive torque

A significant interaction effect was also revealed for peak passive torque (*F*_5,85_ = 6.938, *P* < 0.001, *η*_*p*_^2^ = 0.290). Simple main effects analyses revealed significant increases (Fig. 3b) in torque after each set in EC_1_ (19.3–30.7% [6.9–12.5 Nm], *d* = 1.16–1.48) when compared to pre-intervention, however no change in torque occurred in SS_1_ (0.4–5.1% [− 3.5 to 1.7 Nm], *d* = − 0.15 to 0.23). Despite the significant interaction effect, no between-condition difference was detected at any timepoint (− 8.0 to 22.9% [− 2.4 to 8.4 Nm], *d* = − 0.38 to 0.21), indicative of a crossover profile. Consequently, data were re-examined using a baseline-adjusted ANCOVA, which revealed significantly greater torque in EC_1_ than SS_1_ after each set (7.5–14.1 Nm, *d* = 1.05–1.49).

#### Muscle–tendon unit stiffness

No significant interaction effect was detected for MTU stiffness (*F*_5,85_ = 1.043, *P* = 0.398, *η*_*p*_^2^ = 0.058), with no main effect of condition (*F*_1,17_ = 0.915, *P* = 0.352, *η*_*p*_^2^ = 0.051) but a significant effect of time (*F*_5,85_ = 3.614, *P* = 0.005, *η*_*p*_^2^ = 0.175). When compared to pre-intervention (data collapsed across conditions), significantly lower MTU stiffness (Fig. 3c) was detected after each set (EC_1_ = − 4.6 to − 10.4% [− 0.34 to − 1.12 Nm°^−1^], *d* = − 0.44 to − 0.65; SS_1_ = − 2.8 to − 7.7% [− 0.29 to − 0.91 Nm °^−1^], *d* = − 0.28 to − 0.54; collapsed = − 4.7 to − 9.7% [− 0.47 to − 1.00 Nm°^−1^], *d* = − 0.47 to − 0.96).

#### Achilles tendon stiffness

**Fig. 4:**
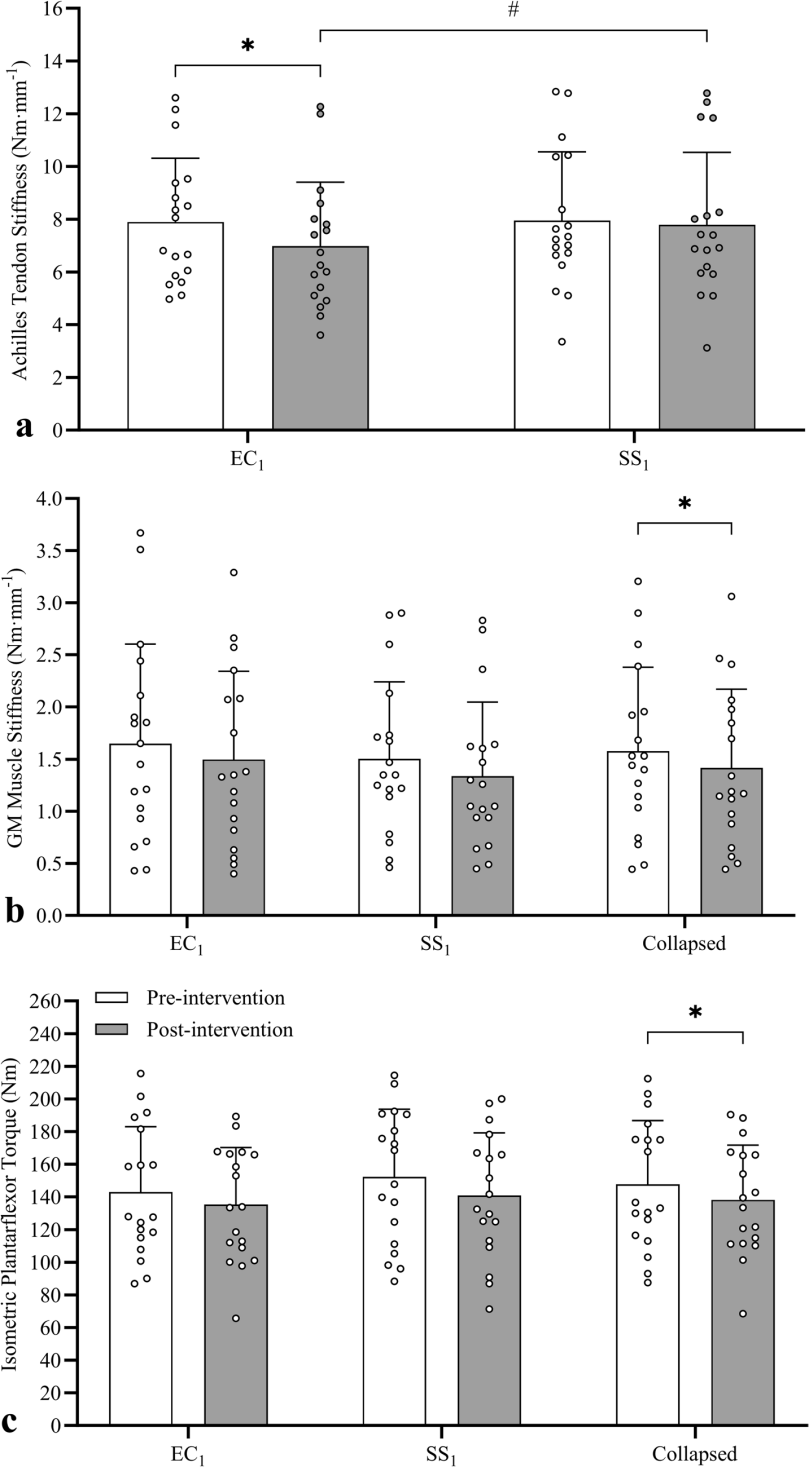
Mean (± SD) and individual Achilles tendon stiffness (**a**), gastrocnemius medialis (GM) muscle stiffness (**b**), and maximal isometric plantarflexor torque (**c**) data measured pre- and post-intervention to detect acute effects during the first eccentric contraction (EC_1_) and static stretching (SS_1_) sessions. Compared to pre-intervention, significant decreases were detected in tendon stiffness only after EC_1_, and in GM muscle stiffness and torque only when data were collapsed. *Significantly different to pre-intervention, ^#^Significant between-condition difference at the same timepoint

A significant interaction effect was found for Achilles tendon stiffness (*F*_1,17_ = 2.520, *P* = 0.043, *η*_*p*_^2^ = 0.220), with simple main effects analyses revealing a significant post-intervention decrease in tendon stiffness (Fig. 4a) in EC_1_ (− 12.0 ± 9.2% [− 0.91 ± 0.79 Nm mm^−1^], *d* = − 1.15) but not SS_1_ (2.1 ± 11.4% [0.16 ± 1.00 Nm mm^−1^], *d* = 0.16). No significant difference was detected between conditions at pre-intervention (2.0 ± 20.0% [0.06 ± 1.32 Nm mm^−1^], *d* = 0.04), however significantly lower tendon stiffness was detected in EC_1_ than SS_1_ at post-intervention (− 8.4 ± 16.2% [− 0.80 ± 1.37 Nm], *d* = − 0.59).

#### Gastrocnemius medialis muscle stiffness

No significant interaction effect was detected for GM muscle stiffness (*F*_1,17_ = 0.028, *P* = 0.869, *η*_*p*_^2^ = 0.002), with no main effect of condition (*F*_1,17_ = 2.028, *P* = 0.173, *η*_*p*_^2^ = 0.107) but a significant effect of time (*F*_1,17_ = 8.521, *P* = 0.010, *η*_*p*_^2^ = 0.334). A significant post-intervention decrease (Fig. 4b) in muscle stiffness (data collapsed across conditions) was detected (EC_1_ = − 6.1 ± 22.2% [− 0.15 ± 0.40 Nm mm^−1^], *d* = − 0.38; SS_1_ = − 10.7 ± 14.5% [− 0.17 ± 0.28 Nm mm^−1^], *d* = − 0.59; collapsed = − 9.2 ± 15.1% [− 0.16 ± 0.27 Nm mm^−1^], *d* = − 0.60).

#### Maximal isometric plantarflexor torque

No significant interaction effect was detected for maximal isometric plantarflexor torque (*F*_1,17_ = 0.281, *P* = 0.603, *η*_*p*_^2^ = 0.016), with no main effect of condition (*F*_1,17_ = 2.087, *P* = 0.167, *η*_*p*_^2^ = 0.109) but a significant effect of time (*F*_1,17_ = 8.521, *P* = 0.010, *η*_*p*_^2^ = 0.334). A significant reduction (Fig. 4c) in torque (data collapsed across conditions) was detected post-intervention (EC_1_ = − 4.5 ± 12.1% [− 11.5 ± 22.3 Nm], *d* = − 0.43; SS_1_ = − 6.5 ± 14.7% [− 7.8 ± 18.3 Nm], *d* = − 0.52; collapsed = − 5.7 ± 10.0% [− 9.6 ± 14.0 Nm], *d* = − 0.69).

### Between-session (carry-over) effects

#### Range of motion

**Fig. 5:**
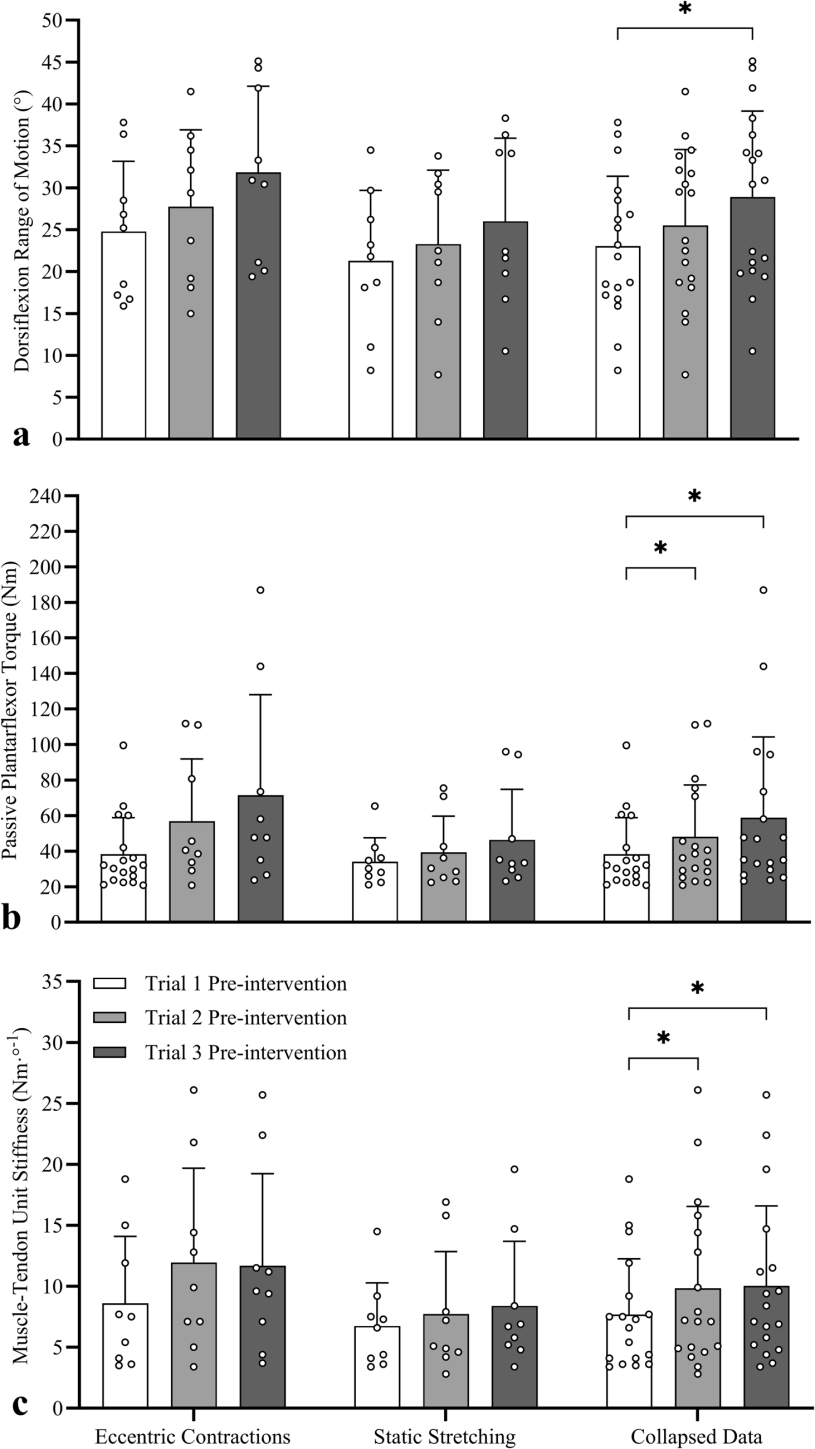
Mean (± SD) and individual dorsiflexion range of motion (**a**), plantarflexor stretch tolerance (**b**), and muscle–tendon unit (MTU) stiffness (**c**) data measured at pre-intervention to detect carry-over effects between Sessions 1, 2, and 3. Compared to Session 1, significant increases were detected in pre-intervention range of motion in Session 3, in stretch tolerance and MTU stiffness in Sessions 2 and 3 when data were collapsed. *Significantly different to pre-intervention in Session 1

To explore whether one or two sessions of eccentric contractions or static stretches were sufficient to increase ROM at baseline in the subsequent sessions (i.e. between-session carry-over effects), the participants were separated into two groups based on whether they performed EC (*n* = 9) or SS (*n* = 9) first. No significant interaction effect was detected for ROM (*F*_1.59,25.38_ = 0.550, *P* = 0.544, *η*_*p*_^2^ = 0.033), with no main effect of condition (*F*_1,16_ = 1.240, *P* = 0.282, *η*_*p*_^2^ = 0.072) but a significant effect of session (*F*_1.59,25.38_ = 13.999, *P* < 0.001, *η*_*p*_^2^ = 0.467). Compared to Session 1 (data collapsed across conditions), no significant ROM increase was detected in Session 2 (collapsed = 2.5° ± 4.5°, *d* = 0.55; EC = 3.0° ± 4.3°, *d* = 0.69; SS = 2.0° ± 5.0°, *d* = 0.40) but a significant increase (Fig. 5a) was detected in Session 3 (collapsed = 5.9° ± 5.9°, *d* = 1.00; EC = 7.1° ± 4.6°, *d* = 1.55; SS = 4.7° ± 7.1°, *d* = 0.67). Thus, a single exposure (training session) was not sufficient to trigger sustained ROM improvement 2–3 days later, but two exposures was sufficient.

#### Peak passive torque

No significant interaction effect was detected for pre-intervention peak passive torque (*F*_1.50,24.06_ = 1.354, *P* = 0.271, *η*_*p*_^2^ = 0.078), with no main effect of condition (*F*_1,16_ = 1.135, *P* = 0.302, *η*_*p*_^2^ = 0.066) but a significant effect of session (*F*_1.50,24.06_ = 12.849, *P* < 0.001, *η*_*p*_^2^ = 0.445). Compared to Session 1 (data collapsed across conditions), significant increases in torque (Fig. 5b) were detected at Session 2 (EC = 34.7 ± 29.3% [14.5 ± 15.2 Nm], *d* = 0.96; SS = 16.4 ± 43.4% [5.4 ± 13.9 Nm], *d* = 0.39; collapsed = 25.4 ± 38.2% [10.0 ± 14.9 Nm, *d* = 0.67) and Session 3 (EC = 62.2 ± 63.4% [29.1 ± 38.9 Nm], *d* = 0.75; SS = 13.9 ± 87.3% [12.3 ± 22.1 Nm], *d* = 0.56; collapsed = 38.0 ± 78.1% [20.7 ± 31.9 Nm], *d* = 0.65). Thus, a single exposure was sufficient to produce a sustained increase in torque 2–3 days later.

#### Muscle–tendon unit stiffness

No significant interaction effect was detected for pre-intervention MTU stiffness (*F*_2,16_ = 0.012, *P* = 1.847, *η*_*p*_^2^ = 0.174), with no main effect of condition (*F*_1,16_ = 1.183, *P* = 0.293, *η*_*p*_^2^ = 0.069) but a significant effect of session (*F*_2,32_ = 8.353, *P* = 0.001, *η*_*p*_^2^ = 0.343). Compared to Session 1 (data collapsed across conditions), significant increases in MTU stiffness (Fig. 5c) were detected at Session 2 (EC = 42.4 ± 46.6% [3.3 ± 3.1 Nm °], *d* = 1.07; SS = 10.8 ± 30.8% [1.0 ± 2.7 Nm °], *d* = 0.37; collapsed = 26.6 ± 41.6% [2.2 ± 3.1 Nm °], *d* = 0.71) and Session 3 (EC = 43.2 ± 58.2% [3.1 ± 3.8 Nm °], *d* = 0.80; SS = 40.2 ± 84.4% [1.7 ± 3.3 Nm °], *d* = 0.50; collapsed = 41.7 ± 70.3% [2.4 ± 3.5 Nm °], *d* = 0.66). Thus, a single exposure was sufficient to produce a sustained increase in MTU stiffness 2–3 days later.

#### Achilles tendon stiffness

**Fig. 6:**
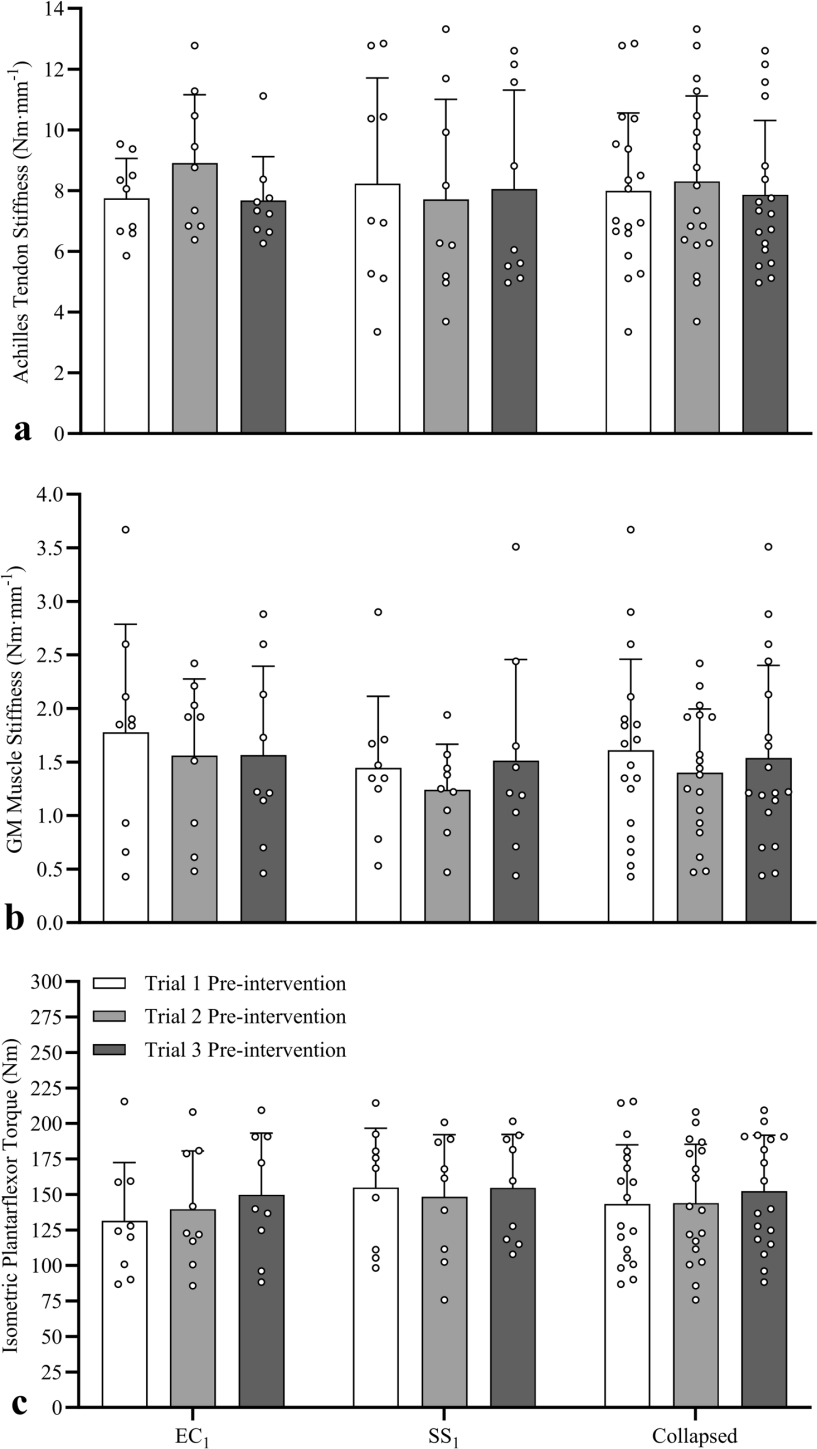
Mean (± SD) and individual Achilles tendon stiffness (**a**), gastrocnemius medialis (GM) muscle stiffness (**b**), and maximal isometric plantarflexor torque (**c**) data measured at pre-intervention to detect carry-over effects between Sessions 1, 2, and 3. No significant changes in pre-intervention Achilles tendon stiffness, GM muscle stiffness, or torque were detected after one or two sessions of eccentric contraction or static stretch sessions

No significant interaction effect (*F*_2,32_ = 3.124, *P* = 0.058, *η*_*p*_^2^ = 0.163) or main effects of condition (*F*_1,16_ = 0.243, *P* = 0.629, *η*_*p*_^2^ = 0.015) or session (*F*_2,32_ = 0.535, *P* = 0.591, *η*_*p*_^2^ = 0.032) were detected for Achilles tendon stiffness (Fig. 6a).

#### Gastrocnemius medialis muscle stiffness

No significant interaction effect (*F*_2,32_ = 0.798, *P* = 0.459, *η*_*p*_^2^ = 0.047) or main effects of condition (*F*_1,16_ = 0.462, *P* = 0.506, *η*_*p*_^2^ = 0.028) or session (*F*_2,32_ = 1.451, *P* = 0.249, *η*_*p*_^2^ = 0.083) were detected for GM muscle stiffness (Fig. 6b).

#### Maximal isometric plantarflexor torque

No significant interaction effect (*F*_1.59,25.46_ = 3.033, *P* = 0.076, *η*_*p*_^2^ = 0.159) or main effects of condition (*F*_1,16_ = 0.418, *P* = 0.527, *η*_*p*_^2^ = 0.025) or session (*F*_1.59,25.46_ = 3.178, *P* = 0.069, *η*_*p*_^2^ = 0.166) were detected for maximal isometric plantarflexor torque (Fig. 6c).

#### Correlations

During SS_1_, no significant within-session correlations were detected between the absolute change in ROM and absolute changes in MTU stiffness (*rs* = 0.25), peak passive torque (*rs* = 0.43), muscle stiffness (*rs* = − 0.20) or tendon stiffness (*rs* = − 0.33), while a significant correlation was detected between changes in MTU stiffness and peak passive torque (*rs* = 0.57). However, during EC_1_ a significant within-session correlation was detected between changes in ROM and peak passive torque (*rs* = 0.66), with no significant within-session correlations detected between the changes in ROM and any other variable (*rs* = − 0.16 to 0.24).

For the baseline assessment across sessions, significant between-session correlations were detected between the changes in ROM and both peak passive torque (*rs* = 0.81–0.85) and MTU stiffness (*rs* = − 0.73–0.80) after one and two sessions of static stretching. However, significant between-session correlations were only found for the changes in ROM and peak passive torque (*rs* = 0.70–0.78) after one and two sessions of eccentric contractions. No significant between-session correlations were detected between the changes in ROM and any other variable after one or two sessions of stretches or eccentric contractions.

## Discussion

The aim of the present study was to examine and compare the within- and between-session dose–response effects of eccentric contractions and static stretches on dorsiflexion ROM. In agreement with our first hypothesis, five sets of eccentric contractions triggered a large increase in dorsiflexion ROM (6.0°), with only a moderate increase detected after static stretches (2.2°). While the increase in ROM after eccentric contractions aligns with the six previous studies assessing their acute effects (Nelson 2006; Nishida et al. 2018; Aune et al. 2019; Kawama et al. 2022, 2023; Muanjai et al. 2022), only one study had directly compared eccentric contractions to static stretching (Nelson 2006). Supporting the second hypothesis, an almost three-fold greater increase in ROM was evoked by eccentric contractions than static stretches, consistent with the almost two-fold greater increase in knee extension ROM (hamstring flexibility) after eccentric contractions (9.5°) versus static stretches (5.5°) observed previously (Nelson 2006). This enhanced efficacy is further substantiated by the between-condition analyses revealing greater ROM after each set of eccentric contraction than static stretching (1.9°–3.9°), with a single set of eccentric contractions achieving similar ROM increases as five sets of static stretches. Collectively, these findings demonstrate superior within-session effectiveness and efficiency of eccentric contractions for inducing acute ROM improvements compared with static stretching, which remains the predominant modality in current practice. Furthermore, as the eccentric contractions were performed through a submaximal ROM, unlike static stretching that requires stretching the muscle through an extreme ROM, these findings may have particular importance for a range of clinical populations that demonstrate impaired ROM (Harvey et al. 2017). However, as static stretching often fails to provide meaningful improvements in these populations (Harvey et al. 2017), caution should be used when attempting to extrapolate the findings of the present study to clinical populations that have already demonstrated differing responses to interventions.

Another important aim was to determine the potential mechanisms underlying any eccentric contraction- or stretch-induced increases in ROM. Whilst literature exists regarding the likely mechanisms underpinning stretch-induced ROM increases (Behm et al. 2016; Shah et al. 2023), limited data exist regarding the changes resulting from eccentric contractions, with only four studies investigating potential mechanisms. These studies reported no changes in stretch tolerance (Nishida et al. 2018; Kawama et al. 2022, 2023) or MTU stiffness (Muanjai et al. 2022), and inconsistent decreases in muscle stiffness across the hamstring muscles (Kawama et al. 2022, 2023). Partially supporting our first hypothesis and the latter findings (Kawama et al. 2022, 2023), significant decreases in muscle and MTU stiffness were detected in both conditions, however significant decreases in Achilles tendon stiffness and increases in stretch tolerance were uniquely observed after eccentric contractions. These findings align with previous reviews reporting stretch-induced ROM increases alongside decreased muscle or MTU stiffness and/or increased stretch tolerance (Behm et al. 2016; Shah et al. 2023), indicating that both mechanical and neurological (perceptual) mechanisms may influence ROM. However, the lack any substantial EMG activity (< 5% MVC), or change in activity, at full ROM confirms that neuromuscular reflexive muscle activity did not influence maximal ROM nor the post-intervention changes in ROM. While the changes in muscle and MTU stiffness and stretch tolerance were also notable after eccentric contractions, additional decreases in tendon stiffness and increases in stretch tolerance were uniquely observed. The significantly greater acute ROM increase after eccentric contractions may be partially explained by these additional adaptations in mechanical properties (tendon stiffness) and neurological characteristics (stretch tolerance or pain perception), likely resulting from the greater loading imposed during eccentric contractions. These higher loads likely influenced tendon stiffness, consistent with previous reports of acute ROM increases and tendon stiffness decreases after maximal isometric contractions (Kay et al. 2015). Speculatively, increased stretch tolerance may be partly explained by enhanced output from Type III muscle afferents during intense contractions (Mazzullo 1978) inhibiting pain perception, as pressure receptors with larger myelinated neurons connect to the same spinal interneurons as unmyelinated nociceptive fibres (Type IV afferents) (Melzack 1993). However, changes in muscle spindle sensitivity, Ia pathway excitability, other afferent pathways (joint receptors, skin stretch or deformation receptors, ligament receptors, etc.) or supraspinal processing may also contribute. Importantly, these are the first data establishing correlations between mechanistic variables and large acute ROM increases after eccentric contractions, providing valuable insights into the mechanisms underlying the almost three-fold greater acute ROM improvement compared to static stretching.

Recent systematic reviews and meta-analyses have documented substantial chronic ROM increases after eccentric training programmes (Vetter et al. 2022; Diong et al. 2022; Kay et al. 2023), with significant increases after only four weeks (Leslie et al. 2017; Geremia et al. 2018; Aune et al. 2019), highlighting the potential for rapid adaptation. Consequently, our final aim was to examine potential between-session carry-over effects to determine whether residual changes could be detected 2–3 days after one or two sessions of each intervention. Due to our counterbalanced design, participants were separated into two groups (EC, *n* = 9; SS, *n* = 9) to remove potential order effects, allowing between-session data from only 9 participants rather than the full 18-participant cohort. Therefore, a potential limitation of this aspect of the study is a smaller sample size limiting our ability to detect between condition effects and these findings should be interpreted cautiously. Significant increases in ROM, MTU stiffness and stretch tolerance were detected in the combined cohort, without significant interaction effects, indicating no statistically significant differences between eccentric contractions and static stretching. Additionally, no changes in muscle or tendon stiffness or maximal isometric torque were detected. The increase in MTU stiffness may be a consequence of exercise-induced muscle damage (likely from the eccentric intervention) influencing tissue stiffness as the mean increases in MTU stiffness were larger after the eccentric (42.4%, *d* = 1.07) than muscle stretching (10.8%, *d* = 0.37). However, no change in passive muscle or active tendon stiffness was detected, and while these findings conflict with our third hypothesis (expecting significant improvements only after eccentric contractions), the reduced sample size may have limited detection of between-condition differences. This proposition is supported by the almost 50% greater mean ROM increase and larger effect size of the change detected after two sessions of eccentric contractions (7.1°, *d* = 1.55) than with moderate changes after static stretches (4.7°, *d* = 0.67). Nonetheless, further research using larger samples is warranted to explore the relative effects of these interventions in early training phases to determine whether eccentric contractions might prove more effective with minimal exposure.

In summary, the almost threefold greater acute ROM increase and 1.5-fold greater carry-over effect after two sessions of eccentric contractions than static stretches demonstrate superior effectiveness of eccentric contractions for improving ankle joint ROM. Importantly, our examination of mechanisms commonly associated with ROM changes revealed a broader adaptive profile after eccentric contractions, likely explaining the enhanced ROM improvements. These findings may have significant practical implications for clinical and rehabilitation settings where isokinetic training is feasible. Similarly, implementing eccentric-dominant resistance training by emphasising the eccentric portion during traditional resistance training could influence current athletic training practices. Finally, given that the eccentric contractions were performed through a submaximal ROM, as numerous clinical populations exhibit impaired ROM with altered neuromuscular and mechanical/architectural properties and reduced responsiveness to static stretching, the present results strongly suggest investigating the impact of eccentric exercise interventions across diverse clinical populations to determine the efficacy of this intervention in populations that can demonstrate disparate responses to physical activity interventions.

## Data Availability

The dataset that supports the findings of this study are openly available from the University of Northampton Research Explorer at 10.24339/88394ecb-7101-4f79-a4b3-2f4f74f5342c.

## Abbreviations

ANCOVA: Analysis of covariance
ANOVA: Analysis of variance
CI: 95% Confidence intervals
CV: Coefficient of variation
*d*: Cohen’s d
EC: Eccentric contractions
GM: Gastrocnemius medialis
ICC: Intraclass correlation coefficient
log_10_: Log transformations
MTJ: Muscle–tendon junction
MTU: Muscle–tendon unit
MVIC: Maximal voluntary isometric contraction
*n*: Number
*η*_*p*_^2^: Partial eta squared
PNF: Proprioceptive neuromuscular facilitation
*r*: Pearson’s product moment correlation coefficient
ROM: Range of motion
*r*_*s*_: Spearman’s rank correlation coefficient
SD: Standard deviation
SPSS: Statistical Package for the Social Sciences
SS: Static stretching
TTL: Transistor-transistor logic
